# Mammalian Community Structure Varies With Distance Between Protected Areas in the Omo Valley, Southwest Ethiopia

**DOI:** 10.1002/ece3.71248

**Published:** 2025-04-23

**Authors:** Tsyon Asfaw, Fikirte Gebresenbet, Claudio Sillero‐Zubiri, Herwig Leirs, Gebremeskel Gizaw, Adane Tsegaye, Wondimu Abate, Hans Bauer

**Affiliations:** ^1^ Evolutionary Ecology Group, Biology University of Antwerp Wilrijk Belgium; ^2^ Department of Natural Resources and the Environment University of New Hampshire Durham New Hampshire USA; ^3^ Wildlife Conservation Research Unit, Department of Biology University of Oxford Oxford UK; ^4^ Ethiopian Wolf Conservation Programme Dinsho Ethiopia; ^5^ Ethiopian Wildlife Conservation Authority Addis Ababa Ethiopia; ^6^ South Nation, Nationalities and People Regional State, Culture and Tourism Office Hawassa Ethiopia

**Keywords:** camera traps, conservation biogeography, large carnivores, mammalian diversity, Omo Valley

## Abstract

Rapid human population growth in Ethiopia has resulted in the degradation of vast areas of wildlife habitats due to agricultural expansion, infrastructure development, and urbanization. The Omo Valley in the southwestern part of Ethiopia has been particularly affected by land use changes, but despite its ecological importance, few relevant studies have been conducted there in the last two decades. Our aim is to provide updated and scientifically verifiable information for medium and large terrestrial mammal species richness and community structure in four Protected Areas in the Omo Valley. We used bycatch camera trap data from a large carnivore survey and nonparametric incidence‐based estimators for data analysis. A total of 52 mammals from nine orders and eighteen families were recorded, of which approximately 29.4% are listed as globally threatened and one as an endemic subspecies. We present the current species lists and compare them with historical records and observed the highest species number in Omo National Park, even though nine species were no longer recorded there. We applied the Morisita‐Horn similarity index to reveal a high degree of overlap in mammalian species among adjacent Protected Areas, but less overlap between Protected Areas far from each other, indicating distance decay of similarity. The distribution of feeding guilds was significantly different across Protected Areas, and carnivore detection frequency was relatively low in Tama Community Conservation Area compared to our other study sites. This study confirmed the conservation importance of the area in terms of mammalian diversity, albeit with low detection levels, especially of large carnivores, underscoring the importance of promoting landscape connectivity to maintain population viability across the Omo Valley. From our experience, the use of camera trap bycatch data proved to be effective in surveying large‐ and medium‐sized mammalian species, but less so in capturing the rarer species in the area.

## Graphical abstract

This study confirmed the conservation importance of the Omo Valley in terms of mammalian diversity, albeit with low detection levels, especially of large carnivores, underscoring the importance of promoting landscape connectivity to maintain population viability across the Omo Valley. Mammalian diversity of the Omo Valley showed a high degree of overlap in mammalian species among adjacent Protected Areas, but less overlap between Protected Areas far from each other, indicating distance decay of similarity. From our experience, the use of camera trap bycatch data proved to be effective in surveying large and medium‐sized mammalian species, but less so in capturing the rarer species in the area.

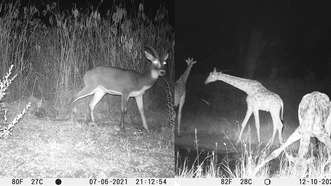

## 1 Introduction

Ethiopia stands out as one of Africa's biodiversity‐rich nations, hosting around 311 mammal species, 55 of them endemic (Lavrenchenko and Bekele 2017; Yalden et al. 1996; Yalden and Largen 1992). Mammalian endemism in Ethiopia is higher than in other African countries, linked to the country's diverse climate and altitudinal range, from 116 m below sea level (Danakil depression) to 4550 m above sea level (Ras Dejen in Simien). Mammalian populations are declining in Ethiopia and across most of Africa due to human pressures (Craigie et al. 2010; Jones et al. 2018). Ethiopia's rapid human population growth leads to the degradation of vast areas of wildlife habitat due to agricultural expansion, infrastructure development, and urbanization (Kelboro and Stellmacher 2015; Wale et al. 2017).

To slow the rate of the current biodiversity loss, IUCN recently proposed the Global Biodiversity Framework to ensure and enable that by 2030 at least 30% of terrestrial, inland water, and coastal and marine areas will be effectively conserved and managed through ecologically representative, well‐connected, and equitably governed systems of Protected Areas or other forms (CBD 2022). The success of Protected Areas in supporting biodiversity conservation then measure through several determinant factors, including species richness, community structure, and the intensity of human activities within the boundaries. Globally, Protected Areas are considered to be a primary defense against biodiversity loss (Rodrigues and Cazalis 2020; Santini et al. 2016; Terraube et al. 2020). Ethiopia has 85 Protected Areas, covering ~192,216 km^2^ (about 17% of the country), most of which are found in southwestern Ethiopia (https://www.protectedplanet.net/country/ETH).

Omo Valley is one of the biodiversity‐rich regions located in the southwest of Ethiopia. Despite the growing human and livestock populations among local pastoral tribes in the Omo Valley, recurring tribal conflicts have inadvertently created a refuge for wildlife (Asfaw et al. 2024). This biologically diverse region supports one of the largest concentrations of wildlife and serves as a corridor for the migration of mammalian populations from Eastern and Central Africa (Asfaw et al. 2022; Ghiglieri 1981; Urban and Brown 1968). Nevertheless, wildlife populations and their habitats in this region have recently been adversely affected by land use changes due to agricultural expansion and development projects, such as the Kuraz Sugar Factory and the Gibe III and IV (Koysha) hydroelectric dams (Yadeta et al. 2022; Armaw and Molla 2022; Jeza and Bekele 2024).

**FIGURE 1:**
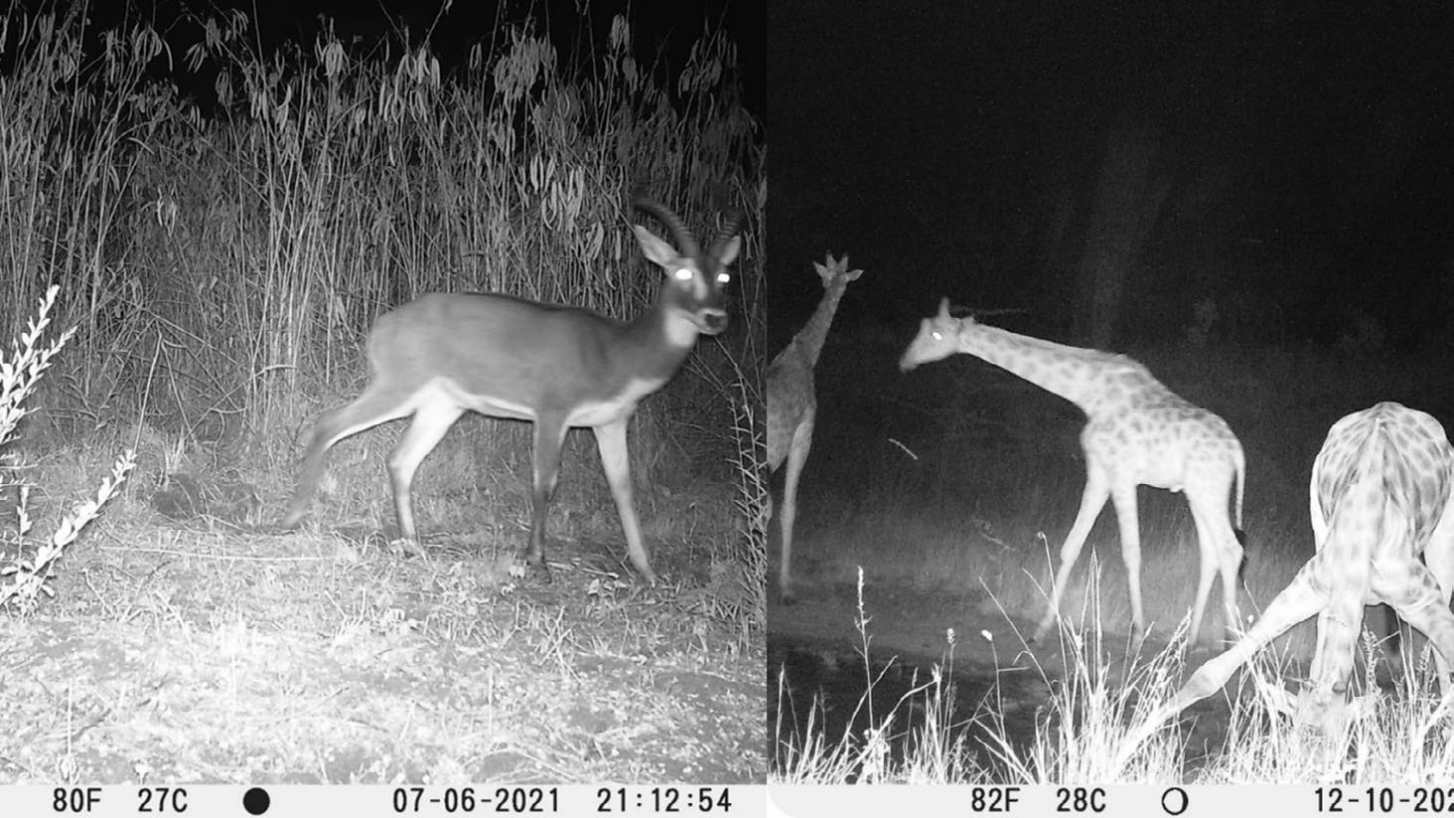
Camera trap image of white‐eared kob from ONP and giraffe from TCCA.

There are precious few studies to illustrate mammal distribution in the Omo Valley (Acha et al. 2017; Ghiglieri 1981; Urban and Brown 1968; Yimer and Yirga 2013). Since Urban and Brown (1968) only scattered data and anecdotal evidence have been produced assessing the potential of the lower Omo Valley in supporting mammalian populations. More recently, Timer (2005) studied the large mammal diversity in Chebera Churchura National Park using transect surveys. Yimer and Yirga (2013) conducted a study on mammalian diversity at Maze National Park using a transect survey. These studies primarily focused on diurnal species, using indirect methods to count nocturnal mammals, including scats, track, and sound (Yimer and Yirga 2013). While they provided valuable insights, transect surveys and indirect methods may not fully capture cryptic, nocturnal, and rare mammalian species (Puri 2011). Our study, therefore, aimed to provide updated and scientifically verifiable information for medium and large terrestrial mammal species richness and community structure in four Protected APreas selected from the Omo Valley based on bycatch camera trap data that were systematically deployed for the purpose of large carnivore surveys (Figure 1).

Mammalian species richness, defined as the number of mammal species in an area, is often used to provide baseline species data at a local scale and evaluate the state of biological diversity at a site (Whittaker 1972). On the other hand, mammalian community structure refers to the composition and organization of mammal species within a particular ecosystem or habitat; and it is crucial for assessing the health and stability of ecosystems and for making informed conservation and management decisions (Begon et al. 2006; Gotelli and Graves 1996). Understanding the species present in an area and the community structure of a site is therefore critical for effectively planning and evaluating biodiversity conservation strategies (Tobler et al. 2008).

## 2 Methods and Methodology

### 2.1 Study Area Description

**FIGURE 2:**
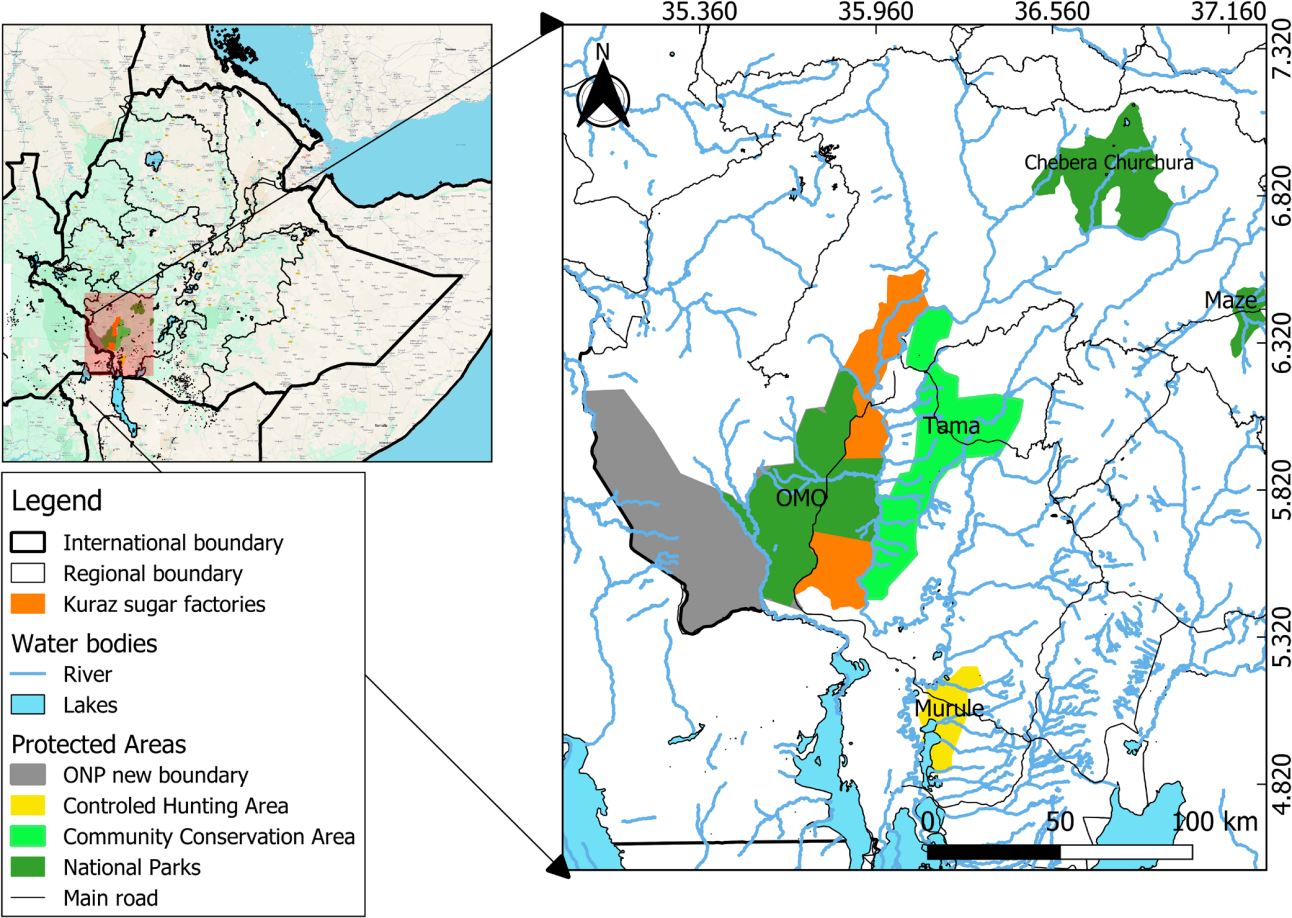
A map showing studied Protected Areas, Murule Control Hunting Area, and the Kuraz Sugar Factory in the Omo Valley, southwest Ethiopia.

The study was conducted in four Protected Areas in the Omo Valley, namely Omo National Park (ONP), Tama Community Conservation Area (TCCA), Maze National Park (MNP), and Chebera Churchura National Park (CCNP) (Figure 2). These Protected Areas are located within the Gibe‐Omo River Basin. Areawise, MNP is the smallest, covering 202 km^2^, while ONP is theoretically the largest site, covering 4068 km^2^. TCCA has a total area of 1968 km^2^ and CCNP extends to 1250 km^2^. The CCNP landscape is characterized by steep slopes, hills, and escarpments, surrounded by highly rugged mountains with altitudinal ranges between 500 to 2000 m asl. On the other hand, the ONP and TCCA landscapes consist of expansive savanna grasslands, hills, and riverine forests along the Omo River. Altitude ranges from 450 to 1183 m asl in the Omo–Tama complex. MNP is characterized by undulating plains and gentle slopes; Maze River and its tributaries flow through the park and drain to the Omo River, creating patches of riverine forest. Altitude ranges between 998 and 1200 m asl in MNP.

The variation in topography influences the rainfall gradient, with the lower lying ONP and TCCA receiving about 780 mm of rainfall per annum, with the main wet season extending from March to November. MNP receives a moderate mean annual rainfall of 975 mm. The rainfall in CCNP ranges from 1200 mm to 2300 mm per annum, with the temperature ranging from 10°C to 29°C. The wet season extends from March to September in both CCNP and MNP. The recorded mean minimum and maximum temperature for ONP and TCCA are 20°C from April to June and 40°C in December, respectively. The mean annual temperature of MNP is 24°C.

**FIGURE 3:**
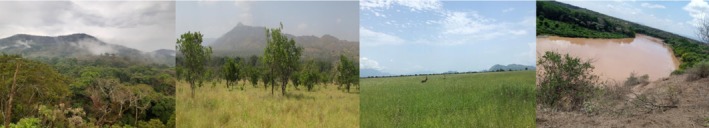
Landscape images of the Protected Areas, showcasing vegetation types and topography. The images, from left to right, depict CCNP, MNP, and the ONP and TCCA complex.

The study areas vary in their vegetation structure, with ONP and TCCA featuring open grasslands, wooded grasslands, forests, and thickets, along with scattered rocky outcrops and hills from which several streams drain into the Omo River (Armaw and Molla 2022). For some analyses, ONP and TCCA were combined into Omo‐Tama Complex (OTC). CCNP contains four major vegetation types: grasslands, woodlands, mountain forests, and riverine forests. MNP is covered by savanna grassland with scattered deciduous broadleaf trees, riverine forest, bushland, and woodland vegetation (Figure 3). Although these Protected APreas are not very close to each other, except for ONP and TCCA, they are connected by rivers that drain into the Omo River (Figure 2).

## 3 Methods

### 3.1 Camera Trap Survey Design

Camera trap surveys for the purpose of the large carnivore study took place from 2020 to 2022 in CCNP, MNP, TCCA, and ONP for a two‐month period each (Asfaw et al. in Press). Due to the large carnivore target, the grid size was informed by the smallest home range sizes for large carnivores in the regions (Asfaw et al. in Press). Thus, we used bycatch camera trap data to assess the mammalian diversity in the region. We used a mixture of Bushnell Trophy Trail Camera 119717cw (Bushnell, California, USA), Minox DTC 550 (Minox, Wetzlar, Germany), Dorr Snapshot Trail Camera (DÖRR, Hamburg, Germany), and Rollei WK cameras (Rollei, Hamburg, Germany). We used Quantum GIS (QGIS) 3.24 Vector Grid Research Tool (Quantum GIS Development Team 2021) to divide each protected area into 25 km^2^ grid squares. The center point of each grid cell was then determined using the QGIS Polygon Centroid Geometry Tool (Quantum GIS Development Team 2021) from which the geographic coordinates were extracted and uploaded onto a handheld GPS device for use in the field. Single unbaited movement‐activated cameras were then placed on trees set alongside roads, drainage lines, signs of the prey species or targeted large carnivores, human and animal trails in the center of the sample grid at 40 cm above the ground but away from anthropogenic activity (Van Berkel 2014). In total, 137 cameras (19 in MNP, 46 in CCNP, 43 in ONP and 43 in TCCA, based on the area coverage of the study site) were placed for a minimum of 60 continuous days and effectively retrieved. The camera trap survey lasted for 10,301 camera days (2799 in ONP; 3089 in TCCA; 717 in MNP and 3696 in CCNP). On placement, we trimmed vegetation and grass, without altering the immediate habitat, within the camera's detection zone to reduce false trigger events.

### 3.2 Data Analysis

All camera trap images were identified to the species level using the Field Guide to African Mammals as a reference (Kingdon 2015); all pictures other than large‐ to medium‐sized mammals were avoided from this study. digikam (https://bugs.kde.org/) and exiftool (https://exiftool.org) programs were used for image tagging and metadata extraction, respectively. Datasets were organized and managed for statistical analysis in R studio using the *camtrapR package* (Niedballa et al. 2016). The images were filtered according to species using 5‐min intervals to ensure independence, and a presence/absence data frame was created for all detected species.

Observed species richness (S_(obs)_), estimated species richness(S_(est)_), and Morisita‐Horn similarity index were analyzed for all studied sites using a nonparametric incidence‐based estimators in the estimates 9.1.0 software (Colwell 2013). The nonparametric approach makes no assumptions about the mathematical form of the underlying distributions of species abundance or species detection rates; it avoids the mathematical drawbacks and is more robust in applications (Chao and Chiu 2016). Thus, we used a nonparametric approach, as our data exhibited a non‐normal distribution due to the rarity of some species and the dominance of others.

We specifically used nonparametric estimators, including Incidence–based Coverage Estimator (ICE), Chao 2, and Jackknife 2, to fit our incidence data, accounting for imperfect detection (Chao and Chiu 2016). The ICE estimator is particularly useful in scenarios where the sampling is incomplete, as it provides a robust estimate of species richness based on the incidence of species presence across samples (Chao and Chiu 2016). Jackknife estimators are useful to estimate species richness from limited or incomplete data by systematically leaving out subsets of the data to estimate richness based on the remaining samples and allowing researchers to derive estimates of species richness without making strong assumptions about the underlying distribution of species abundances (Foggo et al. 2003; Walther and Moore 2005; Saunders 2018). The Morisita‐Horn similarity index assesses the similarity of species composition between different communities, with a focus on understanding the overlap in species between different sampling sites (Chao et al. 2004). This similarity index accounts for both species' presence and abundance; hence, it is a robust choice for comparing communities with varying species richness and abundance distributions (Chao et al. 2004). All these estimators extrapolate species data to the presumed asymptote reflecting the predicted species richness (Gotelli and Graves 1996). The estimators use information on the frequency of rare species in a sample to estimate the number of undetected species, based on the concept that rare species carry the most information about the number of undetected species (Gotelli and Graves 1996). Observed species richness was also presented as the actual number of species captured during the camera trap survey. The Morisita‐Horn index was used to analyze the degree of overlap in species assemblage among Protected Areas (Magurran 2004).

We followed the nomenclature of the IUCN Red List, and body weight data was derived from Kingdon (2015). To understand the community structure, we evaluated how different species contribute to the ecosystem in terms of abundance and biomass across various feeding groups and size classes. The Relative Abundance Index (RAI) was used to estimate the biomass contribution of different herbivore groups within the Protected Areas, and species were grouped by their feeding guilds (i.e., carnivores, herbivores, insectivores, and omnivores) and sizes. RAI is a measure of how often species are detected by camera traps, and this method is commonly used to evaluate the community structure based on camera traps (Reece et al. 2021). Except for species richness estimators, all statistical analysis was implemented in R Studio.

## 4 Results

A total of 137 cameras deployed in four selected Protected Areas from Omo Valley with the mean distance of 4.3 km in ONP, 4.5 km in MNP, and 4.23 km in CCNP between camera traps. These camera traps detected a total of 52 mammalian species from nine orders and eighteen families across the four Protected Areas in the Omo Valley. Overall, 29.4% of the species were listed as globally threatened by the IUCN. An endemic subspecies Swayne's hartebeest ( *Alcelaphus buselaphus swaynei* ) was recorded in MNP.

The highest number of species was observed in ONP (41 species), followed by TCCA (32 species), CCNP (29), and MNP (22). However, the variation in species richness among the four Protected Areas were not significantly different from each other (*p* > 0.05). The highest detection frequency was recorded in CCNP (3158 independent detections of 29 medium and large mammal species) followed by TCCA with 2779 independent detections of 32 species, ONP with 1770 independent detections of 41 species and MNP with 1016 independent detection of 22 species (details and scientific names in Table 1).

**TABLE 1:** Medium and large mammal species detected during the surveys.

Order	Family	Common name (Scientific name)	TCCA			CCNP			ONP			MNP		
			a	b	c	a	b	c	a	b	c	a	b	c
Carnivores			47			112			166			12		
Medium														
Carnivora	Canidae	Black‐backed jackal (*Lupulella mesomelas*)	15	1	0.5	0	0	0.00	9	2	0.32	0	0	0.00
Carnivora	Felidae	Afro‐Asiatic wildcat ( *Felis lybica* )^*VU*^	0	0	0.00	0	0	0.00	8	4	0.29	0	0	0.00
Carnivora	Felidae	Caracal ( *Caracal caracal* )	1	1	0.03	0	0	0.00	3	2	0.11	0	0	0.00
Carnivora	Felidae	Serval ( *Leptailurus serval* )	2	1	0.06	2	1	0.05	32	11	1.14	8	5	1.12
Carnivora	Herpestidae	Common slender mongoose (*Herpestes sanguineus*)	2	1	0.06	11	8	0.30	12	8	0.43	0	0	0.00
Carnivora	Hyaenidae	Aardwolf ( *Proteles cristata* )	0	0	0.00	1	1	0.03	0	0	0.00	0	0	0.00
Large														
Carnivora	Felidae	Lion ( *Panthera leo* )^*VU*^	DO	0	0.00	DO	0	0.00	10	2	0.36	9	4	1.26
Carnivora	Felidae	Cheetah ( *Acinonyx jubatus* )	0	0	0.00	0	0	0.00	6	2	0.21	0	0	0.00
Carnivora	Felidae	Leopard ( *Panthera pardus* )^*VU*^	2	2	0.06	38	10	1.03	43	11	1.54	2	2	0.28
Carnivora	Hyaenidae	Spotted hyaena ( *Crocuta Crocuta* )	25	10	0.81	60	7	1.62	37	9	1.32	1	1	0.14
Carnivora	Hyaenidae	Striped hyaena ( *Hyaena hyaena* )^*NT*^	0	0	0.00	0	0	0.00	5	3	0.18	0	0	0.00
Carnivora	Canidae	African wild dog ( *Lycaon pictus* )^*EN*^	0	0	0.00	0	0	0.00	1	1	0.04	0	0	0.00
Herbivores			1613			1363			1224			446		
Browser														
Primates	Cercopithecidae	Guereza ( *Colobus guereza* )	0	0	0.00	2	2	0.05	1	1	0.04	22	6	3.07
Cetartiodactyla	Bovidae	Guenther's dik‐dik ( *Madoqua guentheri* )	347	1	11.2	0	0	0.00	176	13	6.29	5	1	0.70
Cetartiodactyla	Bovidae	Common duiker ( *Sylvicapra grimmia* )	333	23	10.8	17	9	0.46	175	15	6.25	0	0	0.00
Hyracoidea	Procaviidae	Bush hyrax ( *Heterohyrax brucei* )	0	0	0.00	1	1	0.03	4	2	0.14	6	2	0.84
Cetartiodactyla	Bovidae	Bushbuck ( *Tragelaphus scriptus* )	97	15	3.14	227	30	6.14	50	6	1.79	87	11	12.13
Rodentia	Hystricidae	Crested porcupine ( *Hystrix cristata* )	124	21	4.01	119	15	3.22	91	11	3.25	51	5	7.11
Cetartiodactyla	Giraffidae	Giraffe ( *Giraffa camelopardalis* )^*VU*^	54	1	1.75	0	0	0.00	2	2	0.07	0	0	0.00
Cetartiodactyla	Bovidae	Greater kudu ( *Tragelaphus strepsiceros* )	0	0	0.00	0	0	0.00	0	0	0.00	16	5	2.23
Cetartiodactyla	Bovidae	Kirk's dik‐dik ( *Madoqua kirkii* )	0	0	0.00	0	0	0.00	73	8	2.61	0	0	0.00
Cetartiodactyla	Bovidae	Lesser kudu ( *Tragelaphus imberbis* )^*NT*^	71	1	2.30	0	0	0.00	252	14	9.00	0	0	0.00
Cetartiodactyla	Bovidae	Waterbuck ( *Kobus ellipsiprymnus* )	38	10	1.23	215	20	5.82	22	2	0.79	116	14	16.18
Cetartiodactyla	Bovidae	Bohor redbuck ( *Redunca redunca* )	0	0	0.00	0	0	0.00	0	0	0.00	DO	0	0.00
Cetartiodactyla	Bovidae	Klipspringer ( *Oreotragus oreotragus* )	0	0	0.00	0	0	0.00	7	2	0.25	0	0	0.00
Grazer														
Cetartiodactyla	Hippopotamidae	Hippopotamus ( *Hippopotamus amphibius* )^*VU*^	0	0	0.00	29	4	0.78	0	0	0.00	0	0	0.00
Cetartiodactyla	Bovidae	White‐eared kob ( *Kobus kob leucotis* )	0	0	0.00	0	0	0.00	1	1	0.04	0	0	0.00
Cetartiodactyla	Bovidae	African buffalo ( *Syncerus caffer* )^*NT*^	90	13	2.91	436	24	11.80	56	9	2.00	0	0	0.00
Cetartiodactyla	Bovidae	Oribi ( *Ourebia ourebi* )	153	13	4.95	0	0	0.00	36	7	1.29	34	11	4.74
Cetartiodactyla	Bovidae	^#^Swayne's hartebeest ( *Alcelaphus buselaphus swaynei* )^*EN*^	0	0	0.00	0	0	0.00	0	0	0.00	91	11	12.69
Cetartiodactyla	Bovidae	Lelwel (*A. b. lelwel*)^*EN*^	96	6	3.11		0	0.00	0	0	0.00	0	0	0.
Cetartiodactyla	Bovidae	Topi ( *Damaliscus lunatus* )^*VU*^	0	0	0.00	0	0	0.00	50	6	1.79	0	0	0.00
Perissodactyla	Equidae	Common zebra ( *Equus quagga* )^*NT*^	208	12	6.73		0	0.00	0	0	0.00	0	0	0.00
Mixed														
Rodentia	Sciuridae	Unstriped ground Squirrel ( *Xerus rutilus* )	1	1	0.03	27	6	0.73	7	3	0.25	0	0	0.00
Rodentia	Sciuridae	Striped ground squirrel ( *Xerus erythropus* )	1	1	0.03	12	5	0.32	14	5	0.50	2	1	0.28
Cetartiodactyla	Bovidae	Grant's gazelle ( *Nanger granti* )	0	0	0.00	0	0	0.00	26	3	0.93	0	0	0.00
Cetartiodactyla	Bovidae	Common eland (*Tragelaphus oryx*)	0	0	0.00	0	0	0.00	41	8	1.46	0	0	0.00
Proboscidea	Elephantidae	African savanna elephant ( *Loxodonta africana* )^*EN*^	0	0	0.00	88	5	2.38	19	6	0.68	0	0	0.00
Omnivores			1085			1646			346			544		
Carnivora	Herpestidae	White‐tailed mongoose ( *Ichneumia albicauda* )	68	1	2.20	190	20	5.14	121	19	4.32	16	8	2.23
Carnivora	Viverridae	African civet ( *Civettictis civetta* )	3	2	0.10	5	5	0.14	19	1	0.68	0	0	0.00
Carnivora	Viverridae	Common genet ( *Genetta genetta* )	275	18	8.90	191	24	5.17	179	16	6.40	33	6	4.60
Carnivora	Mustelidae	Honey badger ( *Mellivora capensis* )	1	1	0.03	5	4	0.14	11	7	0.39	0	0	0.00
Cetartiodactyla	Suidae	Bushpig ( *Potamochoerus larvatus* )	32	1	1.04	323	25	8.74	0	0	0.00	56	5	7.81
Cetartiodactyla	Suidae	Giant forest hog ( *Hylochoerus meinertzhageni* )	5	1	0.16	138	15	3.73	0	0	0.00	0	0	0.00
Cetartiodactyla	Suidae	Common warthog ( *Phacochoerus africanus* )	583	29	18.87	317	20	8.58	61	7	2.18	144	13	20.08
Primates	Cercopithecidae	De Brazza's monkey ( *Cercopithecus neglectus* )	5	1	0.16	26	7	0.70	0	0	0.00	2	1	0.28
Primates	Cercopithecidae	Grivet monkey ( *Chlorocebus aethiops* )	0	0	0.00	14	6	0.38	26	2	0.93	30	7	4.18
Primates	Cercopithecidae	Olive baboon ( *Papio anubis* )	77	2	2.49	625	32	16.91	49	9	1.75	279	10	38.91
Primates	Cercopithecidae	Patas monkey ( *Erythrocebus patas* )^*NT*^	36	8	1.17	2	1	0.05	1	1	0.04	0	0	0.00
Insectivores			28			21			8			4		
Tubulidentata	Orycteropodidae	Aardvark ( *Orycteropus afer* )	17	7	0.55	6	3	0.16	2	1	0.07	4	2	0.56
Pholidota	Manidae	Ground pangolin (*Smutsia temminckii*)^*VU*^	2	2	0.06	DO	0	0.00	0	0	0.00	0	0	0.00
Carnivora	Herpestidae	Common dwarf mongoose ( *Helogale parvula* )	9	6	0.29	15	7	0.41	6	3	0.21	0	0	0.00

*Note:* Species are arranged according to feeding strategy with (a) the number of independent detections, (b) number of camera locations a species was detected at, and (c) species capture frequency. IUCN red list status denoted as ^NT^ near threatened, ^VU^ vulnerable ^EN^ endangered, the remaining species listed as least concern. # denotes an endemic subspecies. DO = observed based on direct observation but not included in the analysis.

The two most frequently recorded species were olive baboon ( *Papio anubis* ) and waterbuck ( *Kobus ellipsiprymnus* ) in MNP; lesser kudu ( *Tragelaphus imberbis* ) and guenther's dik‐dik ( *Madoqua guentheri* ) in ONP; guenther's dik‐dik ( *Madoqua guentheri* ) and common duiker ( *Sylvicapra grimmia* ) in TCCA; and African buffalo ( *Syncerus caffer* ) and bushbuck ( *Tragelaphus scriptus* ) in CCNP. Spotted hyaena ( *Crocuta Crocuta* ) was the species caught least frequently, with a single detection in MNP. African wild dog ( *Lycaon pictus* ), guereza ( *Colobus guereza* ), white‐eared kob ( *Kobus kob leucotis* ), patas monkey ( *Erythrocebus patas* ), and aardvark ( *Orycteropus afer* ) were each recorded only in one camera trap location, with a single detection in ONP. On the other hand, caracal ( *Caracal caracal* ) and the two squirrel species ( *Xerus rutilus* and *Xerus erythropus* ) recorded only in one camera trap location, with a single detection in TCCA, while the same is true for aardwolf ( *Proteles cristata* ) and bush hyrax ( *Heterohyrax brucei* ) in CCNP (Table 1).

### 4.1 Species Richness Estimators

Higher estimates than the observed richness were produced across all Protected Areas using the nonparametric species richness estimator. However, Chao 2 produced approximately similar estimates of 42.45 species in ONP and 30.61 in ICE (30.61) (Table 2), each increased by 1.5.

**TABLE 2:** Observed (actual) and predicted (non‐parametric estimators) species richness estimates across four Protected Areas in the Omo Valley.

Protected Apreas	Cumulative trap days	Species richness estimators			
		Observed	ICE	Chao 2	Jack 2
TCCA	3089	32	40.55	47.54	45.48
ONP	2799	41	44.55	42.45	44.28
CCNP	3696	29	30.61	34.83	36.67
MNP	717	21	26.61	30.83	30.67

The Morisita‐Horn similarity measurement index revealed a relatively high degree of overlap in mammalian species among the nearest Protected Areas (CCNP and MNP; ONP and TCCA) and a low degree of overlap among the Protected Areas that are far from each other (Table 3).

**TABLE 3:** Summary of species similarity indices for mammals across the four studied Protected Areas in the Omo Valley.

Protected Areas		Actual observed species		Similarity index estimators		
		1st Sample	2nd Sample	Shared S (obs)	Morisita‐Horn	Bray‐Curtis
TCCA	CCNP	32	29	22	0.453	0.374
TCCA	ONP	32	41	25	0.638	0.483
TCCA	MNP	32	21	16	0.434	0.280
CCNP	ONP	29	41	23	0.359	0.329
CCNP	MNP	29	21	17	0.796	0.392
ONP	MNP	41	21	18	0.252	0.273

*Note:* Values of both Morisita‐Horn and Bray‐Curtis indices range from 0 to 1 meaning no overlap to complete overlap and identical communities to completely dissimilar communities, respectively.

Our camera trap surveys failed to record seven species previously found in the OTC (although two were recorded outside the study sites, that is, in and around Murule Control Hunting Area and Kibish), nine in MNP, and eleven in CCNP. Conversely, three new species were added to the species record list of the MNP and six in the CCNP (Table 4).

**TABLE 4:** Emergence and disappearance of medium to large mammal species in the Omo Valley from historical records available from Urban and Brown (1968), Timer (2005), and Yimer and Yirga (2013).

Order	Family	Species Common Name (Scientific Name)	CCNP	MNP	OTC
Primates	Cercopithecidae	Yellow baboon ( *Papio cynocephalus* )	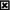	_	_
Primates	Cercopithecidae	Patas monkey ( *Erythrocebus patas* )^*NT*^	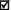	_	_
Primates	Cercopithecidae	De Brazza's monkey ( *Cercopithecus neglectus* )	_	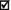	_
Cetartiodactyla	Bovidae	Common duiker ( *Sylvicapra grimmia* )	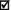	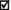	_
Cetartiodactyla	Bovidae	Lesser kudu ( *Tragelaphus imberbis* )^*NT*^	_	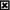	_
Cetartiodactyla	Suidae	Giant forest hog ( *Hylochoerus meinertzhageni* )	_	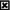	_
Cetartiodactyla	Bovidae	African buffalo ( *Syncerus caffer* )^*NT*^	_	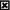	_
Cetartiodactyla	Bovidae	Klipspringer ( *Oreotragus oreotragus* )	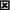	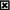	_
Cetartiodactyla	Bovidae	White‐eared kob ( *Kobus kob leucotis* )	_	_	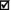
Cetartiodactyla	Bovidae	Greater kudu ( *Tragelaphus strepsiceros* )	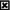	_	_
Cetartiodactyla	Bovidae	^#^Swayne's hartebeest ( *Alcelaphus buselaphus swaynei* )^*EN*^		_	_
Cetartiodactyla	Bovidae	Beisa oryx ( *Oryx beisa* )^EN^	_	_	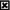
Cetartiodactyla	Bovidae	Tiang ( *Damaliscus korrigum* )	_	_	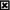
Cetartiodactyla	Bovidae	Gerenuk ( *Litocranius walleri* )^#^	_	_	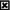
Cetartiodactyla	Bovidae	Mongalla gazelle (*G. thomsoni albonotatus*)	_	_	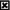
Perissodactyla	Rhinocerotidae	Black rhinoceros ( *Diceros bicornis* )^EN^	_	_	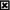
Lagomorpha	Leporidae	African rabbit ( *Lepus victoriae* )	_	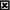	_
Primates	Galagidae	Senegal galago (*Galago senegalensis*)	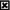	_	_
Rodentia	Sciuridae	Unstriped ground Squirrel ( *Xerus rutilus* )	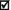	_	_
Rodentia	Sciuridae	Gambian sun squirrel (*Helosciurus gambianus*)	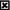	_	_
Carnivora	Mustelidae	Zorilla ( *Ictonyx striatus* )	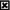	_	_
Carnivora	Canidae	Black‐backed jackal (Lupulella *mesomelas*)	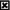	_	_
Carnivora	Canidae	African wild dog ( *Lycaon pictus* )^*EN*^	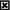	_	_
Carnivora	Canidae	Bat‐eared fox (*Octocyon megalotis*)^#^	_	_	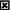
Carnivora	Canidae	African jackal ( *Canis aureus* )	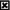	_	_
Carnivora	Canidae	Side‐striped jackal (*Lupulella adustus*)	_	_	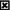
Carnivora	Canidae	Common Jackal	_	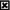	_
Carnivora	Felidae	Caracal ( *Caracal caracal* )	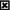	_	_
Carnivora	Hyaenidae	Aardwolf ( *Proteles cristata* )	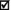	_	_
Carnivora	Hyaenidae	Striped hyaena ( *Hyaena hyaena* )^*NT*^	_	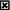	_
Carnivora	Herpestidae	Common slender mongoose (Herpestes sanguineus)	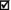	_	_
Carnivora	Herpestidae	Common dwarf mongoose ( *Helogale parvula* )	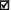	_	_
Carnivora	Viverridae	Common genet ( *Genetta genetta* )	_	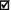	_
Carnivora	Viverridae	Abyssinian genet ( *Genetta abyssinica* )^*DD*^	_	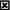	_
Carnivora	Viverridae	African civet ( *Civettictis civetta* )	_	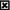	_

*Note:* Observations outside the study sites indicated by # (i.e., Murule Controlled Hunting Area and around Kibish river—Bauer et al. 2024). IUCN Red list status denoted as ^NT^ Near Threatened, ^VU^ Vulnerable ^EN^ Endangered, ^DD^ data deficient the remaining species listed as Least Concern. # denotes an endemic subspecies. 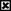 Indicate disappearance. 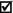 Indicate emergence.

### 4.2 Community Structure

**FIGURE 4:**
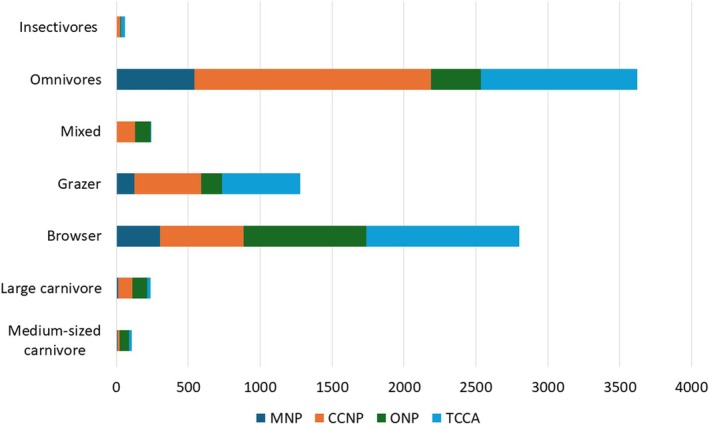
Mammalian community structure based on the detection frequency of different feeding guilds across study sites in the Omo Valley.

Overall, the medium to large mammal community in the Omo Valley was predominantly composed of herbivores, with 25 species, followed by carnivores (12 species), omnivores (11 species), and insectivores (3 species) (Table 1). However, the detection frequency of these feeding guilds showed a statistically significant difference across Protected Areas, indicating the distribution of feeding guilds is dependent on Protected Area (*X* ^2^ = 1211.8, df = 18, *p* < 0.001). For instance, omnivores were the most frequently encountered group in CCNP, whereas in ONP and TCCA, herbivores were more commonly detected. Large carnivores ranked third in detection frequency across all Protected Areas, and higher in ONP followed by CCNP (Figure 4).

Large carnivores were the most frequently encountered carnivores across all Protected Areas, with six species and 239 total detections. Carnivore diversity varied between Protected Areas. ONP had the highest diversity, with six large and five medium‐sized carnivores, accounting for 166 independent detections. CCNP followed, with three large and two medium‐sized carnivores and 112 independent detections. TCCA, which supports two large‐sized and four medium‐sized carnivores with 47 independent detections, was third in large carnivore diversity. MNP supports the lowest diversity, with three large and one medium‐sized carnivore and 20 independent detections (Figure 4).

**FIGURE 5:**
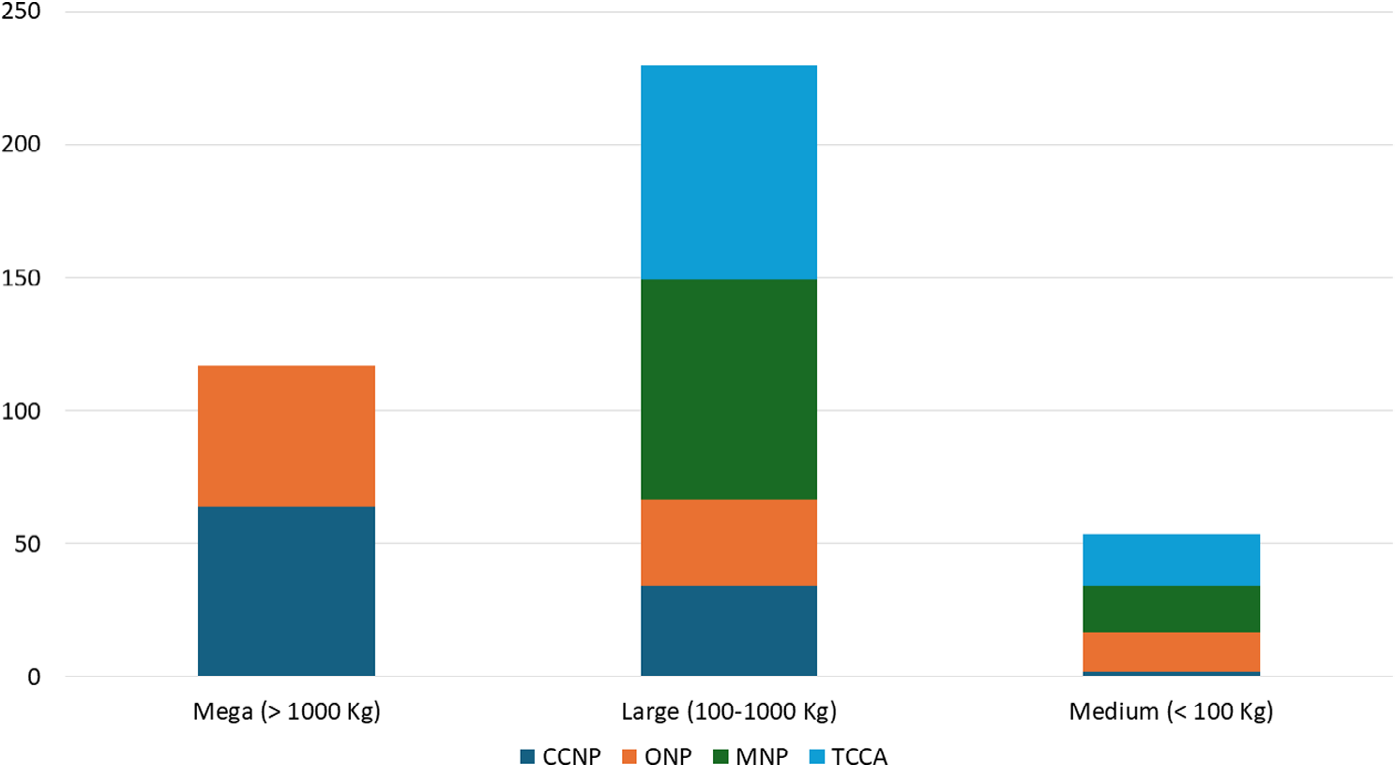
Total herbivore biomass contribution, expressed as a percentage where the number of independent detections per species was used as the basis for biomass contribution calculations.

Among herbivores, browsers were the most frequently encountered, with 12 species accounting for 1737 detections. This was followed by grazers, comprising seven species and 733 detections, and mixed feeders, with seven species and 236 detections. However, when considering biomass contribution, mixed feeders emerged as the highest contributors to the overall medium and large herbivore biomass in both CCNP and ONP, followed by grazers and browsers. In contrast, at MNP, browsers dominated the biomass contribution, accounting for 65.7%, with grazers contributing 34.3%, and mixed feeders contributing only 1% (Figure 5).

Additionally, mega herbivores contributed to 63.7% and 52.7% of the herbivore biomass in CCNP and ONP, respectively, followed by large herbivores at 34% and 33%, and medium herbivores at 2.3% and 14.5%. In MNP and TCCA, where no mega herbivores were present, large herbivores accounted for most of the biomass contribution (Supplementary S1).

## 5 Discussion

The presence of 52 mammalian species (41 from ONP, 32 from TCCA, 29 from CCNP and 22 from MNP) underscores the Omo Valley's rich mammalian diversity. This highlights Omo Valley as an important hotspot for mammalian conservation in Ethiopia, making it a key focus for biodiversity studies and conservation efforts. The presence of endemics and a relatively high number of vulnerable and endangered species highlights the importance of this landscape for conserving and maintaining threatened species, warranting conservation priority. The species richness of ONP alone is comparable with a study in species‐rich African savannah in Serengeti, Tanzania, where 40 mammalian species were found (Swanson et al. 2015). When all the Omo Protected Areas studied are pooled together, the species richness surpasses that of the Serengeti. Similar comparisons can be made with studies conducted in Zimbabwe (Welch et al. 2019). However, the low detection frequency of most species in Omo would suggest that wildlife populations there are likely to be present at low densities.

Nonparametric species richness estimators showed a higher number of species than the observed species across all the study sites. These estimators use the frequency of rare species to estimate the number of undetected species (Gotelli and Graves 1996). Thus, the higher species richness estimated by nonparametric estimators suggested that some species may have been undetected during the survey. However, Chao 2 and ICE estimators showed slightly similar numbers of species with observed species richness in ONP and CCNP than Jackknife. Chao 2 is incidence‐based and uses the ratio of species found in only one sample over the species found in two or more samples (Chao and Chiu 2016), making it sensitive to sampling effort and rare species. On the other hand, ICE divides species into “frequent” and “infrequent” groups based on their presences across sampling sites and calculates richness primarily from the “infrequent” species. This estimator is strongly influenced by species sporadically occurring across samples. Thus, a larger sample size is required for reliable estimates. Whereas the Jackknife focuses on the frequency of species observed only once (singletons) or twice (doubletons) in the samples and is sensitive to species that appear rarely in the dataset but not to the total sample size. Thus, the slightly similar estimates by Chao 2 and ICE indicated the reliability of the sample size and sampling effort, while Jackknife highlighted the limited capacity of camera trap surveys in detecting rare species. Previous studies conducted in the region also suggested the limitation of camera trap surveys and call‐ups in surveying rare species such as lions, cheetahs, and African wild dogs and recommended incorporating additional approaches for future work (Asfaw et al. in Press). This is further supported by the study that indicated the effectiveness of combined monitoring techniques allowing the identification of high mammalian species with low density, larger home range size, and cryptic behaviors (Verschueren et al. 2025; Hausser et al. 2017).

Our records showed changes in species compositions from historical records, despite not capturing all species believed to be present in the area. These species observation differences in our findings could be attributed to various factors, including differences in survey methodologies (Timer 2005; Urban and Brown 1968; Yimer and Yirga 2013), rarity of the species (O'Brien 2008), shift in habitat/range extension (Asfaw et al. 2022), or potential local extinctions. O'Brien (2008) cautioned against drawing conclusions from comparisons of richness estimates with historical species lists, as these lists may not accurately reflect the current species assemblage of an area. This was especially true for rare species like lion and ground pangolin that were not detected by camera trap but that were observed directly and indirectly. During our surveys, we directly observed a lion cub in TCCA and found signs of lion presence in CCNP as well as a direct observation in CCNP after the surveys, suggesting that the absence of lions and certain other species in the camera trap surveys might be due to limitations of the methods in detecting rare species rather than a true absence. Therefore, combining camera trap survey with other methods, including environmental DNA (Bertola et al. 2022); collaring, and/or spatially explicit capture‐recapture (Royle and Young 2008; Shams et al. 2024); walking line transects, vehicle transects, opportunistic encounters and indirect signs (Hausser et al. 2017) and sniffing dog (Verschueren et al. 2025), would be beneficial for future wildlife inventories, in order to improve detection and refine biodiversity assessments. We consider the local extinction of black rhinoceros ( *Diceros bicornis* ) in Omo quite likely, as no reliable observations have been reported since the records of Urban and Brown (1968). Our study highlights ongoing challenges and emphasizes the urgent need for effective conservation measures in Omo. Regular inventory surveys of the area are required for understanding population trends and the role of the region in conservation efforts. The recent finding of white‐eared kob ( *Kobus kob leucotis* , Lichtenstein and Peters 1853) in ONP further strengthens the importance of regular inventories to understand the current situation of the area and its role in landscape connectivity. White‐eared kob is known to occur in the Gambela‐Boma landscape in western Ethiopia and South Sudan. In Omo National Park, one of our camera traps took two images of a white‐eared kob, suggesting that its range extends further to Omo than previously known; therefore, the entire area (Gambella to Omo) can be considered as a range extension (Asfaw et al. 2022).

Our study supported the explanation by Nekola and White (1999) of the distance decay of similarity in biogeography and ecology. Even though the Omo Valley is often referred to as a single ecosystem, our Morisita‐Horn similarity measurement index showed similarity of species composition within the two sets of areas close to each other but divergence between the Protected Areas that are far from each other. This showed that the ecosystem is not uniform, and that there was a decline in ecological similarity between communities as the geographical distance between them increased (Nekola and White 1999). This phenomenon is fundamental in understanding biodiversity patterns driven by either a decrease in environmental similarity with distance (e.g., soil type, moisture, vegetation type, or land use), or by limits to dispersal and niche width differences among taxa. Over larger distances, factors such as habitat fragmentation, climatic differences, and human‐induced disturbances might contribute to divergence in species assemblages (McKinney and Lockwood 1999; Nekola and White 1999; Asfaw et al. in Press). In the case of the Omo Valley, wildlife populations and their habitats have been adversely affected by a combination of both land use land cover changes and human wildlife conflict (Armaw and Molla 2022; Asfaw et al. in Press). To foster the movement of wildlife, especially the movement of wide‐ranging species within the Omo Valley region, conservation planning should aim to promote connectivity between the Protected Areas in the Omo Valley. Vegetation structure plays a significant role in shaping mammalian diversity and community structure (Brophy and McDonald 2019; Rovero et al. 2014), but we did not have sufficient data on vegetation. Thus, future studies that incorporate vegetation type analysis would enhance our understanding of how habitat characteristics shape community composition in these regions.

From a community structure perspective, our study is similar to a study in Majete Wildlife Reserve, in southern Malawi in the Miombo Woodland Ecoregion (Reece et al. 2021), where herbivores made up the largest component of the medium to large mammal community followed by carnivores and omnivores. In the Omo Valley mega and larger herbivores predominantly contributed to biomass, as in Majete. There is a marked difference, however, when it comes to guilds; in Majete, grazers alone contributed 47% of the total biomass (Reece et al. 2021). In ONP and CCNP, the mixed feeders dominate the herbivore community while browsers are dominant in MNP. While camera traps can establish the relative importance of guilds, they cannot, of course not provide the abundance information required to make absolute inferences.

Given the high level of herbivore detection, carnivore detection frequency was relatively low in TCCA, compared to our other studied sites including ONP, CCNP, and MNP. This confirms that prey availability alone does not determine the abundance of large carnivores; and that anthropogenic disturbance is an essential factor (Asfaw et al. in Press). This is further supported by multiple studies that highlighted how human disturbance such as habitat destruction, hunting, poaching, and conflict with humans limit large carnivore populations more than prey availability does (Ripple et al. 2014; Balme et al. 2010; Loveridge et al. 2016; Crooks 2002; Woodroffe and Ginsberg 1998). In general, this study highlights how the use of modern technology, such as camera traps, has proven effective in surveying large and medium‐sized mammalian species. It also showed their limited effectiveness in capturing the rarest species, suggesting species richness can be inferred from camera traps, but caution is needed when making comparisons with historical records. Besides, the results from the Jackknife species richness estimator indicated the effectiveness of camera traps in detecting rare species is determined by the duration of camera traps deployment. This is further supported by Tobler et al. (2008) and Burton et al. (2015), who emphasized the importance of survey effort in determining species detection rates. Therefore, future studies should consider extending camera traps deployment across multiple seasons to build a more comprehensive temporal dataset. This approach might improve detection rates, especially for mammalian species with low density, wide home range sizes, and elusive behaviors.

## Author Contributions

**Tsyon Asfaw:** conceptualization (lead), data curation (lead), formal analysis (lead), funding acquisition (equal), investigation (lead), methodology (lead), project administration (equal), resources (equal), validation (equal), visualization (lead), writing – original draft (lead), writing – review and editing (lead). **Fikirte Gebresenbet:** funding acquisition (equal), project administration (equal), resources (equal), supervision (equal), validation (equal), writing – review and editing (equal). **Claudio Sillero‐Zubiri:** funding acquisition (equal), project administration (equal), resources (equal), supervision (equal), validation (equal), writing – review and editing (equal). **Herwig Leirs:** conceptualization (supporting), project administration (equal), supervision (equal), validation (equal), writing – review and editing (equal). **Gebremeskel Gizaw:** data curation (equal), investigation (equal), validation (equal). **Adane Tsegaye:** data curation (equal), investigation (equal), validation (equal). **Wondimu Abate:** data curation (equal), investigation (equal), validation (equal). **Hans Bauer:** conceptualization (equal), data curation (equal), funding acquisition (equal), project administration (equal), resources (equal), supervision (lead), validation (equal), writing – review and editing (equal).

## Ethics Statement

While no formal ethical approval process exists for this type of research in Ethiopia, appropriate ethical considerations were undertaken throughout the study. Permission for conducting the research was obtained following the review and approval of the study proposal by the Ethiopian Wildlife Conservation Authority and the South Nation, Nationalities, and Peoples' Region authorities. We ensured that all camera trap images unrelated to mammals were excluded from the analysis to maintain privacy and ethical standards. The research was conducted in compliance with national laws and regulations, and efforts were made to minimize any disturbance to wildlife and local communities.

## Conflicts of Interest

The authors declare no conflicts of interest.

## Supporting information


**Supinfo S1.** Biomass Contribution of Mega‐, Large‐, and Medium‐Sized Herbivores in the Four Protected Areas of Omo Valley.

## Data Availability

All the data used in this study are available in the Dryad data repository at http://datadryad.org/stash/share/WTo81OAu7om50NFRsvpHEkV1BIR0YGlOq0BSVBxHevc.
