# Supplementary material for: Mammalian Community Structure Varies With Distance Between Protected Areas in the Omo Valley, Southwest Ethiopia
Source: Ecol Evol. 2025 Apr 23;15(4):e71248. doi: 10.1002/ece3.71248 (PMC12018531; doi:10.1002/ece3.71248)
Supplement: Supplementary file 1 — Supinfo S1. Biomass Contribution of Mega‐, Large‐, and Medium‐Sized Herbivores in the Four Protected Areas of Omo Valley. [file ECE3-15-e71248-s001.docx]

**Supplementary 1: Biomass Contribution of Mega, Large and Medium Sized Herbivores in the Four PAs of Omo Valley.**

| **Order** | **Family** | **Species common name(latin name)** | **CCNP** | | | | **ONP** | | | | **MNP** | | | | **TCCA** | | | |
| --- | --- | --- | --- | --- | --- | --- | --- | --- | --- | --- | --- | --- | --- | --- | --- | --- | --- | --- |
| ***Herbivores*** |  |  | **Total Biomass** | **Weight** | **Independent detection** | **Camera traps** | **Total Biomass** | **Weight** | **Independent detection** | **Camera traps** | **Total Biomass** | **Weight** | **Independent detection** | **Camera traps** | **Total Biomass** | **Weight** | **Independent detection** | **Camera traps** |
| ***Medium*** |  |  |  |  |  |  | **31435** |  |  |  | **8725** |  |  |  | **25510** |  |  |  |
| Primates | Cercopithecidae | Guereza *(*Colobus guereza*)* | 10 | 5 | 2 | 2 | 5 | 1 | 1 | 0.04 | 110 | 22 | 6 | 3.07 | 0 | 0 | 0 | 0 |
| Cetartiodactyla | Bovidae | Guenther's Dik-dik *(Madoqua guentheri****)*** | 0 | 4 | 0 | 0 | 704 | 176 | 13 | 6.29 | 20 | 5 | 1 | 0.7 | 1388 | 347 | 1 | 11.2 |
| Cetartiodactyla | Bovidae | Common Duiker *(Sylvicapra grimmia)* | 255 | 15 | 17 | 9 | 2625 | 175 | 15 | 6.25 | 0 | 0 | 0 | 0 | 4995 | 333 | 23 | 10.8 |
| Hyracoidea | Procaviidae | Bush Hyrax *(Heterohyrax brucei)* | 3 | 3 | 1 | 1 | 12 | 4 | 2 | 0.14 | 18 | 6 | 2 | 0.84 | 0 | 0 | 0 | 0 |
| Cetartiodactyla | Bovidae | Bushbuck *(Tragelaphus scriptus)* | 18160 | 80 | 227 | 30 | 4000 | 50 | 6 | 1.79 | 6960 | 87 | 11 | 12.13 | 7760 | 97 | 15 | 3.14 |
| Rodentia | Hystricidae | Crested Porcupine *(Hystrix cristata)* | 1785 | 15 | 119 | 15 | 1365 | 91 | 11 | 3.25 | 765 | 51 | 5 | 7.11 | 1860 | 124 | 21 | 4.01 |
| Cetartiodactyla | Bovidae | Kirk's Dik-dik *(Madoqua kirkii****)*** | 0 | 6 | 0 | 0 | 438 | 73 | 8 | 2.61 | 0 | 0 | 0 | 0 | 0 | 0 | 0 | 0 |
| Cetartiodactyla | Bovidae | Lesser Kudu *(Tragelaphus imberbis)* | 0 | 80 | 0 | 0 | 20160 | 252 | 14 | 9 | 0 | 0 | 0 | 0 | 5680 | 71 | 1 | 2.3 |
| Cetartiodactyla | Bovidae | Klipspringer *(Oreotragus oreotragus)* | 0 | 15 | 0 | 0 | 105 | 7 | 2 | 0.25 | 0 | 0 | 0 | 0 | 0 | 0 | 0 | 0 |
| Cetartiodactyla | Bovidae | White-eared kob *(Kobus kob leucotis)* | 0 | 60 | 0 | 0 | 60 | 1 | 1 | 0.04 | 0 | 0 | 0 | 0 | 0 | 0 | 0 | 0 |
| Cetartiodactyla | Bovidae | Oribi *(Ourebia ourebi)* | 0 | 25 | 0 | 0 | 900 | 36 | 7 | 1.29 | 850 | 34 | 11 | 4.74 | 3825 | 153 | 13 | 4.95 |
| Rodentia | Sciuridae | Unstriped ground Squirrel (*Xerus rutilus*) | 27 | 1 | 27 | 6 | 7 | 7 | 3 | 0.25 | 0 | 0 | 0 | 0 | 1 | 1 | 1 | 0.03 |
| Rodentia | Sciuridae | Striped Ground Squirrel *(Xerus erythropus)* | 12 | 1 | 12 | 5 | 14 | 14 | 5 | 0.5 | 2 | 2 | 1 | 0.28 | 1 | 1 | 1 | 0.03 |
| Cetartiodactyla | Bovidae | Grant's Gazelle *(Nanger granti)* | 0 | 40 | 0 | 0 | 1040 | 26 | 3 | 0.93 | 0 | 0 | 0 | 0 | 0 | 0 | 0 | 0 |
| **Large** |  |  | **304600** |  |  |  | **70700** |  |  |  | **41500** |  |  |  | **104800** |  |  |  |
| Cetartiodactyla | Giraffidae | Giraffe *(Giraffa camelopardalis)* | 0 | 800 | 0 | 0 | 1600 | 2 | 2 | 0.07 | 0 | 0 | 0 | 0 | 43200 | 54 | 1 | 1.75 |
| Cetartiodactyla | Bovidae | Greater Kudu *(Tragelaphus strepsiceros)* | 0 | 120 | 0 | 0 | 0 | 0 | 0 | 0 | 1920 | 16 | 5 | 2.23 | 0 | 0 | 0 | 0 |
| Cetartiodactyla | Bovidae | Waterbuck *(Kobus ellipsiprymnus)* | 43000 | 200 | 215 | 20 | 4400 | 22 | 2 | 0.79 | 23200 | 116 | 14 | 16.18 | 7600 | 38 | 10 | 1.23 |
| Cetartiodactyla | Bovidae | Common Eland *(T*ragelaphus oryx) | 0 | 600 | 0 | 0 | 24600 | 41 | 8 | 1.46 | 0 | 0 | 0 | 0 |  |  |  |  |
| Cetartiodactyla | Bovidae | Swayne’s Hartebeest *(Alcelaphus buselaphus swaynei*) | 0 | 180 | 0 | 0 | 0 | 0 | 0 | 0 | 16380 | 91 | 11 | 12.69 | 0 | 0 | 0 | 0 |
| Cetartiodactyla | Bovidae | Topi *(Damaliscus lunatus)* | 0 | 130 | 0 | 0 | 6500 | 50 | 6 | 1.79 | 0 | 0 | 0 | 0 | 0 | 0 | 0 | 0 |
|  |  | lelwel |  |  |  |  |  |  |  |  |  |  |  |  | 0 | 96 | 6 | 3.11 |
|  |  | Common Zebra |  |  |  |  |  |  |  |  |  |  |  |  | 0 | 208 | 12 | 6.73 |
| Cetartiodactyla | Bovidae | African Buffalo *(*Syncerus caffer) | 261600 | 600 | 436 | 24 | 33600 | 56 | 9 | 2 | 0 | 0 | 0 | 0 | 54000 | 90 | 13 | 2.91 |
| ***Mega*** |  |  | **571500** |  |  |  | **114000** |  |  |  | **0** |  |  |  | 0 |  |  |  |
| Cetartiodactyla | Hippopotamidae | Hippopotamus *(Hippopotamus amphibius)* | 43500 | 1500 | 29 | 4 | 0 | 0 | 0 | 0 | 0 | 0 | 0 | 0 | 0 | 0 | 0 | 0 |
| Proboscidea | Elephantidae | African Savanna Elephant *(Loxodonta africana)* | 528000 | 6000 | 88 | 5 | 114000 | 19 | 6 | 0.68 | 0 | 0 | 0 | 0 |  |  |  |  |
|  |  |  | 876100 | 0 | 0 | 0 | **216135** |  |  |  | **50225** |  |  |  | **130310** |  |  |  |
|  |  | Mega | 65.23227942 |  |  |  | 52.74481227 |  |  |  | 0 |  |  |  | 0 |  |  |  |
|  |  | Large | 34.76772058 |  |  |  | 32.71103708 |  |  |  | 82.62817322 |  |  |  | 80.42360525 |  |  |  |
|  |  | Medium | #VALUE! |  |  |  | 14.54415065 |  |  |  | 17.37182678 |  |  |  | 19.57639475 |  |  |  |
